# Twice-Daily Theta Burst Stimulation of the Dorsolateral Prefrontal Cortex Reduces Methamphetamine Craving: A Pilot Study

**DOI:** 10.3389/fnins.2020.00208

**Published:** 2020-03-25

**Authors:** Di Zhao, Yongqiang Li, Ting Liu, Valerie Voon, Ti-Fei Yuan

**Affiliations:** ^1^Shanghai Key Laboratory of Psychotic Disorders, Shanghai Mental Health Center, Shanghai Jiao Tong University School of Medicine, Shanghai, China; ^2^Rehabilitation Medicine Center, The First Affiliated Hospital of Nanjing Medical University, Nanjing, China; ^3^School of Psychology, Nanjing Normal University, Nanjing, China; ^4^Department of Psychiatry, University of Cambridge, Cambridge, United Kingdom; ^5^Co-innovation Center of Neuroregeneration, Nantong University, Nantong, China

**Keywords:** addiction, transcranial magnetic stimulation, theta burst stimulation, craving, DLPFC (dorsolateral prefrontal cortex)

## Abstract

**Objectives:**

Transcranial magnetic stimulation (TMS) holds potential promise as a therapeutic modality for disorders of addiction. Our previous findings indicate that high-frequency repetitive transcranial magnetic stimulation (rTMS) over the left dorsal–lateral prefrontal cortex (DLPFC) and low-frequency rTMS over the right DLPFC can reduce drug craving for methamphetamine. One major issue with rTMS is the duration of treatment and hence potential dropout rate. Theta burst stimulation (TBS) has been recently shown to be non-inferior relative to repetitive transcranial magnetic stimulation for major depression. Here, we aim to compare the clinical efficacy and tolerability of intermittent and continuous theta burst stimulation protocols targeting left or right dorsolateral prefrontal cortex on methamphetamine craving in abstinent-dependent subjects.

**Methods:**

In this randomized single-blind pilot study, 83 abstinent methamphetamine-dependent subjects from a long-term residential treatment program were randomly allocated into three groups: intermittent theta burst stimulation (iTBS) over the left DLPFC (active group), continuous theta burst stimulation (cTBS) over the left DLPFC (active control group), or cTBS over the right DLPFC (active group) was administered twice daily over 5 days for a total of 10 sessions. We measured the primary outcome of cue-induced craving and secondarily sleep quality, depression, anxiety, impulsivity scores, and adverse effects.

**Results:**

We show a pre- vs. postintervention effect on craving, which, on paired *t* tests, showed that the effect was driven by iTBS of the left DLPFC and cTBS of the right DLPFC, reducing cue-induced craving but not cTBS of the left DLPFC. We did not show the critical group-by-time interaction. The secondary outcomes of depression, anxiety, and sleep were unrelated to the improvement in craving in the left iTBS and right cTBS group. In the first two sessions, self-reported adverse effects were higher with left iTBS when compared to right cTBS. The distribution of craving change suggested greater clinical response (50% improvement) with right cTBS and a bimodal pattern of effect with left iTBS, suggesting high interindividual variable response in the latter.

**Conclusion:**

Accelerated twice-daily TBS appears feasible and tolerable at modulating craving and mood changes in abstinent methamphetamine dependence critically while reducing session length. We emphasize the need for a larger randomized controlled trial study with a sham control to confirm these findings and longer duration of clinically relevant follow-up.

**Clinical Trial Registration:**

Chinese Clinical Trial Registry number, 17013610.

## Introduction

Disorders of addiction, or compulsive drug-seeking behaviors despite adverse negative consequences, are characterized by abnormal brain network function (Goldstein and Volkow, 2011; Everitt and Robbins, 2016). Preclinical and translational studies highlight a prominent role for hypoactivity of the prefrontal cortex (PFC) with chronic stimulant exposure, leading to the hypothesis that potentiation of PFC function with brain stimulation might improve addiction management (Diana et al., 2017). In the recent decade, non-invasive, repetitive transcranial magnetic stimulation (rTMS) over the dorsolateral prefrontal cortex (DLPFC) has been used to treat cue-induced craving or drug intake across different types of drug dependence, including methamphetamine, cocaine, and heroin (Shen et al., 2016; Terraneo et al., 2016; Su et al., 2017). A range of prefrontal neural regions have been targeted with rTMS including superior frontal gyrus (Rose et al., 2011) or medial prefrontal cortex (Hanlon et al., 2017), whereas we focus here on DLPFC targeting. Convergent evidence has suggested that facilitating the left DLPFC or inhibiting the right DLPFC may reduce craving and substance consumption in patients with substance dependence (Zhang et al., 2019). High-frequency excitatory rTMS of the left DLPFC has been reported to be effective in cocaine use disorder [e.g., 15 Hz/8 sessions/100% motor threshold (Terraneo et al., 2016), 15 Hz/10 sessions/100% motor threshold (Politi et al., 2008), and 10 Hz/single session/90% motor threshold (Camprodon et al., 2007)] and nicotine use disorder [e.g., high frequency/13 session/120% motor threshold, deep TMS over bilateral lateral prefrontal and insula (Dinur-Klein et al., 2014), 10 Hz/10 sessions/100% motor threshold, and 20 Hz/8 sessions/110% over the DLPFC (Amiaz et al., 2009; Sheffer et al., 2018)]. Other stimulants such as methamphetamine craving similarly decreased with high-frequency left DLPFC rTMS (10 Hz/5 sessions/80% motor threshold) (Su et al., 2017), but with enhanced cue craving observed with low frequency (1 Hz/single session/100% motor threshold) (Li et al., 2013b). In heroin-dependent subjects, high-frequency rTMS of the left DLPFC similarly decreased craving (10 Hz/5 sessions/100% motor threshold) (Shen et al., 2016). In contrast, alcohol-dependent subjects showed a different response as a function of laterality with decreased craving with high-frequency rTMS of the right DLPFC (10 Hz/10 sessions/110% motor threshold) (Mishra et al., 2010), with no effects on craving in female alcoholics with high-frequency rTMS of the left DLPFC (20 Hz/10 sessions/110% motor threshold) (Höppner et al., 2011). The rTMS protocol is commonly administered for up to 10–30 min/day with treatment duration lasting between 20 and 30 days. Critically, as treatment compliance is a major issue in drug addiction, decreasing the duration of treatment might enhance the likelihood of completed treatment. Here, we focus on shorter stimulation protocols to reduce session lengths and visits that might lead to improved accessibility for non-invasive neuromodulation for addiction management.

Intermittent or continuous theta burst stimulation (iTBS or cTBS) are TMS protocols that have been shown to, respectively, enhance or inhibit local brain regional activity with long-lasting effect (Huang et al., 2005; Suppa et al., 2016). The protocols involve 600 pulses and requires 3 min for iTBS and 40 s for cTBS (Huang et al., 2005). Previous studies have demonstrated that iTBS has shown comparable neurophysiological excitatory effects to 10 Hz rTMS (Di Lazzaro et al., 2005; Lopez-Alonso et al., 2014). Continuous TBS for 10 sessions to the medial prefrontal cortex has shown potential efficacy for cocaine use disorder (Hanlon et al., 2017). Recently, iTBS was shown in a randomized trial of major depression to be non-inferior to the 10 Hz rTMS protocol in reducing depressive symptoms with similar tolerability and safety profiles (Blumberger et al., 2018). Moreover, iTBS showed similar efficacy to 10 Hz rTMS but, given its shorter duration, might allow a 10-fold increase in the number of patients treated in cocaine use disorder (Sanna et al., 2019). Preliminary studies in major depression have also reported that twice-daily rTMS appears feasible, tolerable, and capable of achieving efficacy similar to once-daily rTMS while reducing treatment course length twofold (McGirr et al., 2015; Modirrousta et al., 2018; Schulze et al., 2018). A recent study has also shown accelerated iTBS as a treatment for cocaine use disorder (Steele et al., 2019). However, no trials have been published to date that explore the feasibility and clinical effects achieved with accelerated (twice-daily) TBS approaches in methamphetamine-dependent patients.

A recent meta-analysis has supported the different left/right hemispheric roles for craving (e.g., cued craving is associated with left DLPFC) and impulsivity (e.g., the suppression of right DLPFC increases the level of impulsive decision making) (Gordon, 2016). Previous studies have suggested that potentiation of the left DLPFC and suppression of the right DLPFC may be effective in reducing cue-induced craving (Li et al., 2013a; Shen et al., 2016; Terraneo et al., 2016; Yavari et al., 2016; Diana et al., 2017). Furthermore, iTBS to the left DLPFC has been shown to produce transsynaptic suppression of the right DLPFC (i.e., the dominant hemisphere in right-handed individuals) via transcallosal connections (George et al., 1999). In the present study, the rationale for choosing iTBS over the left DLPFC and cTBS over the right DLPFC is supported by the above-mentioned studies. We hypothesized that iTBS-L DLPFC and cTBS-R DLPFC would demonstrate efficacy in improving craving symptoms and that cTBS-L DLPFC might act as an active control with an increase in craving symptoms. We further included other secondary outcome measures to assess the role of potential confounders given the known effects of neuromodulation of the DLPFC on mood and impulsivity measures. Critically, we hypothesized that a twice-daily TBS would be feasible in methamphetamine use disorder. We further compared tolerability and self-reported adverse events across different sessions of treatment and among the three accelerated TBS protocols.

## Materials and Methods

### Human Subjects

All the participants were right-handed male, 18–60 years old, and recruited from a long-term residential treatment center. Inclusion criteria included those whose main diagnosis was methamphetamine use disorder with a duration of at least 1 year and using more than 0.1 g a day for at least 3 months. Subjects had a positive urine drug screening test upon admission to a long-term residential treatment program. Subjects could use other substances before admission but must have had only methamphetamine use disorder as their primary addiction diagnosis (except nicotine use disorder). The diagnosis of moderate–severe methamphetamine use disorder was confirmed by a senior psychiatrist [Diagnostic and Statistical Manual of Mental Disorder, Version V (DSM-V)]. The psychiatrist ruled out other severe psychiatric disorders including schizophrenia, bipolar disorder, or severe major depression. Exclusion criteria included a history of other psychiatric disorders, epilepsy, cardiovascular complications, and other contraindications to TMS (e.g., metal implants in the skull). Subject characteristics and previous methamphetamine use history is reported in Table 1. In the rehabilitation center, all participants received standardized rehabilitation including daily physical exercise, supportive therapy on relapse prevention, but no medications. As the rehabilitation program is an enforced residential drug treatment program, participants maintained abstinence in the study. Ethics approval was granted by the Research Ethics Boards of Shanghai Mental Health Center, Nanjing Normal University and the local safety monitoring board (Chinese Clinical Trial Registry number, 17013610). All participants provided written informed consent in accordance with the Declaration of Helsinki.

**TABLE 1:** Demographic and clinical characteristics of patients.

	iTBS-L-D (*n* = 26)	cTBS-L-D (*n* = 18)	cTBS-R-D (*n* = 30)	*F*	*P* value
Age (years)	31.30 (9.60)	29.50 (5.50)	28.23 (6.24)	1.19	0.31
Education (years)	8.54 (2.45)	9.50 (1.90)	8.18 (1.95)	2.16	0.12
Number of cigarettes smoked/day	8.88 (6.10)	7.33 (2.4)	8.20 (6.08)	0.44	0.65
Duration of meth use (years)	6.50 (3.71)	5.78 (3.39)	6.80 (3.29)	0.49	0.61
Duration of current abstinence (months)	6.80 (5.20)	7.89 (6.70)	5.43 (4.20)	1.29	0.28
Meth use before abstinence (g/month)	18.80 (8.89)	23.88 (7.96)	22.48 (14.03)	1.47	0.24
Interval between admission into the rehabilitation center and entry into the study (days)	82.35 (62.99)	101.28 (59.48)	99.37 (71.80)	0.615	0.54
Baseline**** Craving	65.19 (22.20)	65.83 (22.44)	74.83 (19.14)	1.77	0.18
Baseline**** PSQI	6.7 (3.1)	7.1 (3.1)	8 (2.7)	1.41	0.25
Baseline**** BDI	13.6 (7.5)	12.8 (7.2)	17.5 (9.4)	2.405	0.1
Baseline**** BAI	25 (4.5)	29.1 (9.98)	29.8 (8.83)	2.79	0.07
Baseline**** BIS-11	80.36 (14.43)	87.22 (14.82)	83.67 (14.07)	1.226	0.30

*Data are given as mean (SD). BDI, Beck Depression Inventory; BAI, Beck Anxiety Inventory; BIS-11, Barratt Impulsiveness Scale 11.*

A total of 83 inpatients were recruited and randomly assigned (with a computer generated number sequence) into iTBS-L DLPFC (*n* = 27), cTBS-L DLPFC (*n* = 26), and cTBS-R DLPFC (*n* = 30) groups. All patients were naive to TMS. Patients recruited in the study did not participate in other intervention studies before. All patients received twice-daily TBS over five consecutive days for a total of 10 sessions. The following were not included in the data set: six subjects were transferred to a different rehabilitation center before study onset (one iTBS-L and five cTBS-L), and three subjects withdrew before study completion (three cTBS-L). There were no significant differences in demographic variables (e.g., age, years of drug abuse history, number of cigarettes smoked per day, monthly dosage, interval between admission into the rehabilitation center and entry into the study, baseline craving, sleep quality, depression, anxiety, and impulsivity) between study completers and non-completers.

### DLPFC-TBS Procedures

TBS was applied with a CCY-I TMS instrument (Yiruide Co., Wuhan, China), using a figure eight or round-shaped coil for targeted stimulation over the left or right DLPFC. The TMS intensity for each individual participant was calculated as 70% of the resting motor threshold. The motor hand area was localized by TMS that evoked responses of the contralateral abductor pollicis brevis (APB) muscle. The resting motor threshold was determined as the TMS intensity that elicited the APB muscle responses in 5 out of 10 TMS pulses, which produced five motor-evoked potentials responses of at least 50 mV in 10 trials (Kammer et al., 2001) (iTBS-L DLPFC, 28% ± 6%; cTBS-L DLPFC, 27% ± 6%; cTBS-R DLPFC, 29% ± 7%). The DLPFC target was located using the Yiruide TMS Location Cap based on the 10–20 electroencephalography (EEG) system [i.e., F3 and F4 localization for the left and right DLPFC, respectively (Herwig et al., 2003)]. The TMS coil was held above the head of participants with a customized coil holder, and the handle of the coil was rotated to a position where the plane of the coil made an angle of 45° relative to the midline, producing a posterior–anterior current flow within underlying cortical areas. The procedure used for iTBS is composed of three pulse trains of 50 Hz at 70% resting motor threshold (MT) (based on pilot study and the tolerance level for most subjects), which was repeated at 5 Hz (2 s on, 8-s interval) for 3 min (600 pulses in total). In the case of cTBS, three pulse trains of 50 Hz at 70% resting MT was repeated at 200 ms for 40 s (600 pulses in total). The interval of time between the two sessions of treatment delivered on the same day was ∼4 h. Baseline craving, quality of sleep, depression, anxiety, and impulsivity were assessed before the first TBS session (pre-TBS) and, on day 6, the day after the final TBS session (post-TBS).

### Blinding

In this randomized, single-blind study, one experimenter (who administered the intervention) was not blinded to the group assignment, while both the participants and another experimenter (the outcome assessor) were blinded. After all treatments, we asked them to guess whether they had received effective or non-effective stimulation and how much they felt stimulation may have affected them [1 (much worse) to 9 (much better)] to monitor effectiveness of the blinding.

### Measurement

The main outcome measure was the craving score evaluation, which was performed as previously described (Shen et al., 2016). The patients watched a video showing methamphetamine intake for 5 min followed by a visual analogue scale (VAS) to evaluate cue-induced craving scores [range: 0 (no desire or wanting) to 100 (very high desire or wanting)]. The same video was used before treatment (pre-TBS) and after 5 days of treatment (post-TBS). Patients were assessed for cued craving score pre- and posttreatment, which was used to categorize subjects as responders or non-responders. For major depression, changes from baseline values were examined for the 17-item Hamilton Rating Scale in two subject groups (responders and non-responders) (Bakker et al., 2015). Response to TMS was defined as 50% symptoms reduction from pre- to posttreatment (Schulze et al., 2018). Similarly, in the present study, a TBS responder was defined as having at least a 50% reduction in cued craving scores post-TBS compared with baseline.

As the mechanism underlying neuromodulation effects targeting the DLPFC may be related to effects on other symptoms, most particularly depression and impulsivity, we also assessed other secondary outcome measures. The 21-item Beck Depression Inventory (BDI) and 21-item Beck Anxiety Inventory (BAI) were used to assess depressive and anxiety symptoms (Beck et al., 1961; Beck et al., 1988). The Pittsburgh Sleep Quality Index (PSQI) was used to assess sleep quality and consists of 19 self-rated items with 7 components: subjective sleep quality, sleep latency, sleep duration, sleep efficiency, sleep disturbance, use of sleep medication, and daytime dysfunction (Buysse et al., 1989). The 30-item Barratt Impulsiveness Scale-11 (BIS-11) is a self-report measure of impulsivity that assesses six different subtypes of impulsivity (attention, motor, self-control, cognitive complexity, perseverance, and cognitive instability impulsiveness) (Reise et al., 2013).

Subjects were assessed for nine adverse reactions after each treatment session including headache, neck pain, scalp pain, tingling, itching, burning sensations, sleepiness, trouble concentrating, and acute mood change. Each item was scored on a scale of 1 (mild)–10 (severe), with the total score recorded as the sum of all nine items. For tolerability comparisons, each patient’s mean self-reported total score across all sessions was calculated.

### Statistical Analysis

The data were assessed for normality of distribution (Kolmogorov–Smirnov test) and outliers. For data that were not normally distributed, non-parametric statistical analyses were used. There were no outliers. Homogeneity of our intervention groups for baseline demographic and clinical characteristics was confirmed. One-way analyses of variance (ANOVAs) or chi-square test were used to compare group differences for continuous or dichotomous variable comparisons, respectively. A two-way repeated measures analysis of variance (RMANOVA) was used to analyze the effects of TBS on our primary outcome of cued craving and also our secondary outcomes of sleep quality, anxiety, depression, and impulsivity between groups, respectively, with time (pre, post) as a within-subject factor and group (iTBS-L DLPFC, cTBS-L DLPFC, and cTBS-R DLPFC) as a between-subjects factor. Two-sided paired *t* tests were performed between conditions when a significant main effect or time × group interaction was observed. Multiple comparisons were corrected using false discovery rate (FDR) correction (Benjamini and Yekutieli, 2001). When we observed a significant TBS effect in craving scores and other clinical indices (sleep quality, depression, anxiety, and impulsivity), Pearson’s correlation was conducted in exploratory analyses for each TBS group separately to test the relationship between the two indices. Multiple comparisons were corrected for pairwise correlations using FDR correction.

In a secondary analysis of the cued craving score, the improvement percentage was calculated as the percentage change between baseline pre- and post-TBS treatment craving scores. Kernel density estimate (KDE), a non-parametric method to estimate the probability density function of a continuous random variable, was then used to model the distribution of craving score changes acting as a continuous replacement for the discrete histogram. The Kolmogorov–Smirnov test showed that the probability distribution was not normally distributed. We then used the non-parametric Wald–Wolfowitz test to compare the distributions between groups.

For adverse effects, the total scores for all nine items (headache, neck pain, scalp pain, tingling, itching, burning sensations, sleepiness, trouble concentrating, and acute mood change) after each treatment were calculated and compared using a two-way RMANOVA with treatment sessions (1–10) as a within-subject factor and group as a between-subjects factor. The total scores for each adverse event were analyzed using one-way ANOVA. Further paired *t* tests were all FDR corrected.

For blinding effectiveness, the self-report ratings after all treatments were compared using the Kruskal–Wallis Test with group as a factor.

All data were analyzed by IBM SPSS Statistics version 20.1 (IBM Inc., New York, NY, United States) and Matlab R2014b (MathWorks, MA, United States) environments. The statistical significance threshold was set at *P* < 0.05 (two-tailed).

## Results

**FIGURE 1:**
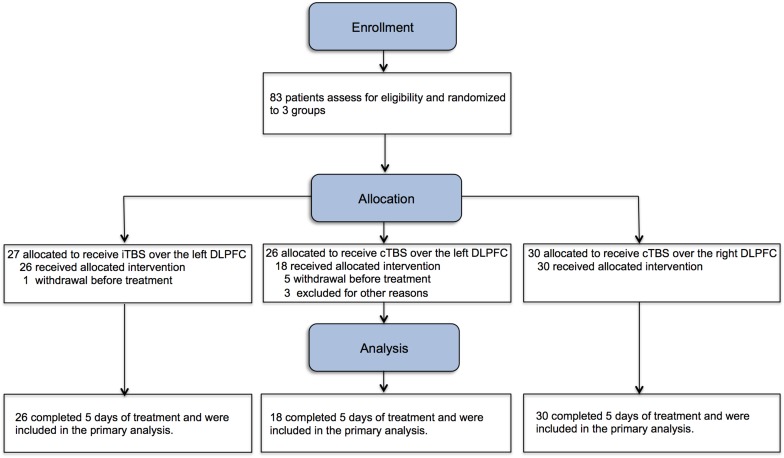
Study flowchart. Eighty-three male methamphetamine-dependent subjects were assigned into three groups for twice-daily theta burst stimulation (TBS) procedures for continuous 5 days (10 sessions). iTBS, intermittent theta burst stimulation; cTBS, continuous theta burst stimulation.

Seventy-four subjects completed 5 days of treatment (iTBS-L DLPFC, *N* = 26; cTBS-L DLPFC, *N* = 18 and cTBS-R DLPFC, *N* = 30) (Figure 1). There were no group differences at baseline in age, years of drug abuse history, number of cigarettes smoked per day, monthly dosage, interval between admission into the rehabilitation center and entry into the study, baseline craving, sleep quality, depression, anxiety, and impulsivity (Table 1).

### Effects of TBS on Craving, Sleep Quality, Mood, and Impulsivity

**FIGURE 2:**
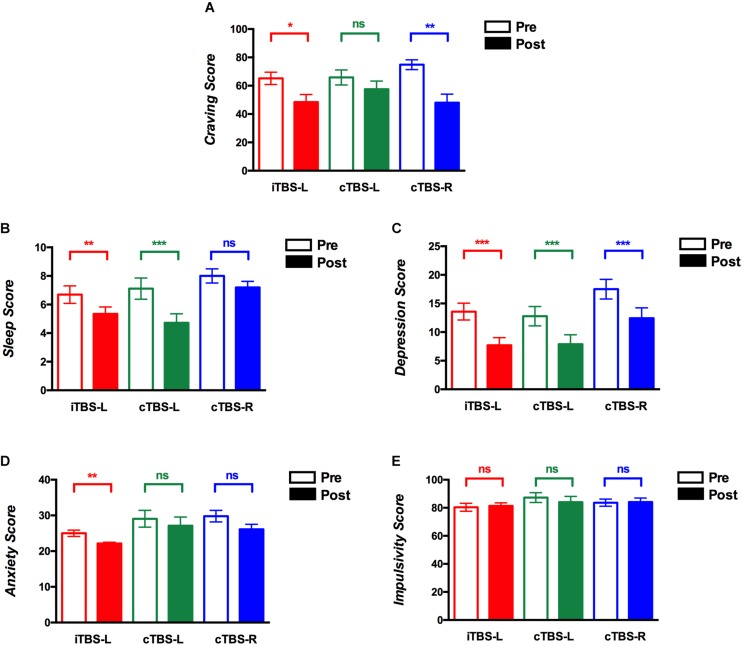
Theta burst stimulation (TBS) intervention on cue-induced craving, quality of sleep, depression, anxiety, and impulsivity scores. The *y*-axis shows mean scores before and after treatment sessions on **(A)** cue-induced craving, **(B)** sleep quality, **(C)** depressive symptoms, **(D)** anxiety symptoms, and **(E)** impulsivity (red, intermittent theta burst stimulation of the left dorsolateral prefrontal cortex (iTBS-L DLPFC); green, continuous TBS of the left DLPFC (cTBS-L DLPFC); blue, continuous TBS of the right DLPFC (cTBS-R DLPFC). Multiple comparisons were corrected using false discovery rate (FDR) correction, *P < 0.05, **P < 0.01, ***P < 0.001; error bars denote SEM).

Table 2 shows the results of ANOVAs conducted for cue-induced craving, sleep quality, mood, anxiety, and impulsivity. The craving score showed a significant main effect of time (*F*_1, 73_ = 21.01, *P* < 0.001, η^2^ = 0.23), suggesting that craving improved pre- vs. post-intervention. To assess the role of specific conditions, two-sided paired *t* tests (FDR corrected) were used to compare craving scores before and after treatment. As shown in Figure 2A, iTBS-L DLPFC (*P* = 0.01) and cTBS-R DLPFC (*P* = 0.001) significantly reduced craving scores but not cTBS-L DLPFC (*P* = 0.52) (Figure 2A). There were no significant main effects of group (*F*_2, 146_ = 0.47, *P* = 0.63, η^2^ = 0.01) nor time × group interaction (*F*_2, 146_ = 2, *P* = 0.14, η^2^ = 0.05).

**TABLE 2:** Results of the ANOVAs conducted for craving, PSQI, BDI, BAI, and BIS.

Measure	Source	*df*	*F*	Sig.	η^2^
Cued-Craving	Time	1	21.01	<0.001	0.23
	Group	2	0.47	0.63	0.01
	Time × group	2	2.00	0.14	0.05
PSQI	Time	1	38.35	<0.001	0.35
	Group	2	3.68	0.03	0.09
	Time × group	2	3.38	0.04	0.09
BDI	Time	1	49.64	<0.001	0.41
	Group	2	0.17	0.84	0.005
	Time × group	2	3.05	0.05	0.08
BAI	Time	1	9.06	0.004	0.11
	Group	2	4.05	0.02	0.10
	Time × group	2	0.27	0.76	0.008
BIS	Time	1	0.18	0.68	0.002
	Group	2	0.75	0.48	0.02
	Time × group	2	0.74	0.48	0.02

*Pittsburgh Sleep Quality Index (PSQI); BDI, Beck Depression Inventory; BAI, Beck Anxiety Inventory; BIS, Barratt Impulsiveness Scale.*

**FIGURE 3:**
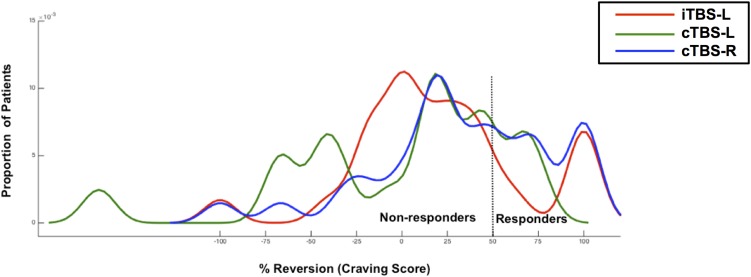
The response distribution curve. Kernel density estimates of cued-craving distributions (shown as percentage improvement from pre- to posttreatment) in methamphetamine-dependent patients (*N* = 74) who were receiving either intermittent TBS of the left dorsolateral prefrontal cortex (iTBS-L DLPFC) (red), or continuous TBS of the left DLPFC (cTBS-L DLPFC) (green) or continuous TBS of the right DLPFC (cTBS-R DLPFC) (blue). A TBS responder was defined as having at least a 50% reduction in cued craving scores post-TBS compared with baseline.

KDE of the distribution functions for percentage improvement (cue-induced craving) from pretreatment for each group is shown in Figure 3. The Kolmogorov–Smirnov test indicated that the distribution of percent change in craving was not normal for iTBS-L DLPFC (*D* = 0.32, *P* = 0.006), cTBS-L DLPFC (*D* = 0.37, *P* = 0.02), and cTBS-R DLPFC (*D* = 0.33, *P* = 0.003). We showed a significant difference in the probability distribution between iTBS-L DLPFC and cTBS-L DLPFC (Wald–Wolfowitz test, *P* = 0.03) with a marginally significant difference between cTBS-L DLPFC and cTBS-R DLPFC (*P* = 0.06). In the cTBS L-DLPFC group, patients’ craving symptoms in three responders (16.67%) improved on average by 45 points (from 65 to 20; 95% CI, −9.1 to 99.1). In contrast, craving scores in 11 responders (36.67%) in the cTBS R-DLPFC group and 6 responders (23.08%) in the iTBS L-DLPFC group demonstrated improvements of 57.3 (from 72.7 to 15.4; 95% CI, 41.9–72.6) and 55 (from 66.7 to 11.7; 95% CI, 40.5–69.5), respectively.

The sleep quality score demonstrated a main effect of time (*F*_1, 73_ = 38.35, *P* < 0.001, η^2^ = 0.35) and group (*F*_2, 146_ = 3.68, *P* = 0.03, η^2^ = 0.09) and a time × group interaction (*F*_2, 146_ = 3.38, *P* = 0.04, η^2^ = 0.09). In the cTBS-L DLPFC (*P* = 0.009, FDR corrected) and iTBS-L DLPFC (*P* = 0.0002, FDR corrected) groups, sleep quality scores showed a significant reduction or improvement with no differences shown with cTBS-R DLPFC (*P* = 0.08, FDR corrected) (Figure 2B). Thus, although sleep quality improved pre- vs. posttreatment, this was driven by the cTBS-L and iTBS-L relative to cTBS-R DLPFC groups.

Repeated measures analysis of variance for depressive symptom scores indicated a main effect of time (*F*_1, 73_ = 49.64, *P* < 0.001, η^2^ = 0.41), suggesting an overall pre- vs. posttreatment improvement. As shown in Figure 2C, compared to baseline, there was a significant decrease in all posttreatment depression scores (iTBS-L DLPFC, *P* = 0.0009; cTBS-L DLPFC, *P* = 0.001; cTBS-R DLPFC, *P* = 0.0005; FDR corrected). Neither a significant main effect of group (*F*_1, 73_ = 0.17, *P* = 0.84, η^2^ = 0.005) nor time × group interaction (*F*_2, 146_ = 3.05, *P* = 0.05, η^2^ = 0.08) was found.

Repeated measures analysis of variance for anxiety scores suggested a main effect of time (*F*_1, 73_ = 9.06, *P* = 0.004, η^2^ = 0.11) and group (*F*_2, 146_ = 4.05, *P* = 0.02, η^2^ = 0.1). In the *post hoc* analysis, only iTBS-L DLPFC showed a decrease in anxiety scores (*P* = 0.001, FDR corrected) (Figure 2D). The interaction of time × group (*F*_2, 146_ = 0.27, *P* = 0.76, η^2^ = 0.008) was not significant.

Repeated measures analysis of variance for impulsivity revealed neither a main effect nor interaction (Table 2 and Figure 2E).

### Correlation Analyses Between Changes Across Different Clinical Indexes

**FIGURE 4:**
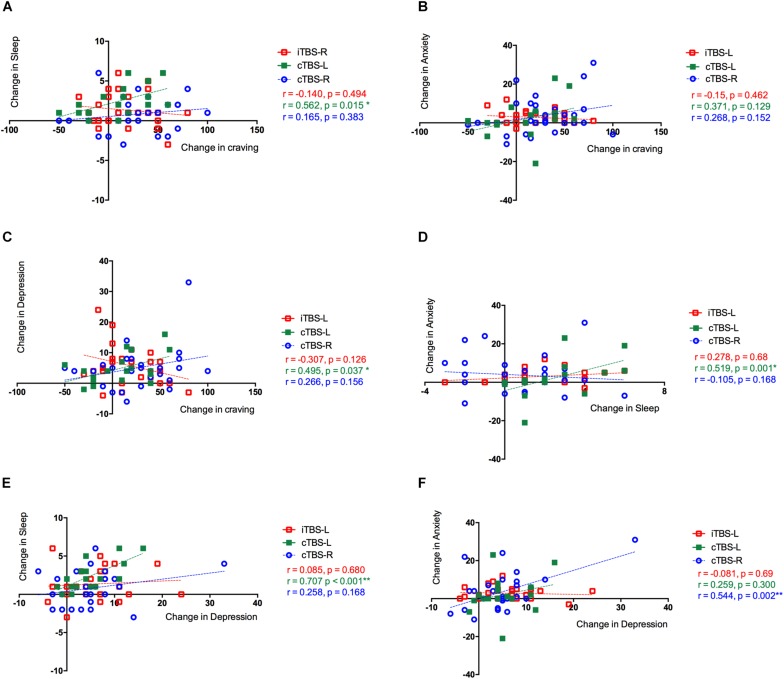
Correlations between changes between clinical outcomes in three groups. The Pearson correlation between changes in **(A)** cue-induced craving and sleep quality, **(B)** cue-induced craving and anxiety level, and **(C)** cue-induced craving and depression level. **(D)** Sleep quality and anxiety level and **(E)** sleep quality and depression level. **(F)** Anxiety level and depression level. The three treatment groups were assessed separately showing the effects of theta burst stimulation (TBS) between clinical symptoms: intermittent TBS of the left dorsolateral prefrontal cortex (iTBS-L DLPFC) (red), continuous TBS of the left DLPFC (cTBS-L DLPFC) (green), or continuous TBS of the right DLPFC (cTBS-R DLPFC) (blue).

We conducted exploratory analyses on the primary outcome measure of craving and secondary outcome measures to assess potential relationships to other clinical outcomes. A positive correlation of changes between craving and sleep quality (*r* = 0.56, *P* = 0.045, FDR corrected) and craving and depression (*r* = 0.495, *P* = 0.037, FDR corrected) was observed in the cTBS-L DLPFC group but critically not in the iTBS-L DLPFC and cTBS-R DLPFC groups, both of which demonstrated significant improvement in craving with intervention. Changes between sleep quality and anxiety in cTBS-L DLPFC was significantly correlated (*r* = 0.519, *P* = 0.003, FDR corrected). In the cTBS-R DLPFC group, changes between depression and anxiety were also significantly correlated (*r* = 0.707, *P* = 0.001, FDR corrected) (Figure 4).

### Adverse Reactions

**FIGURE 5:**
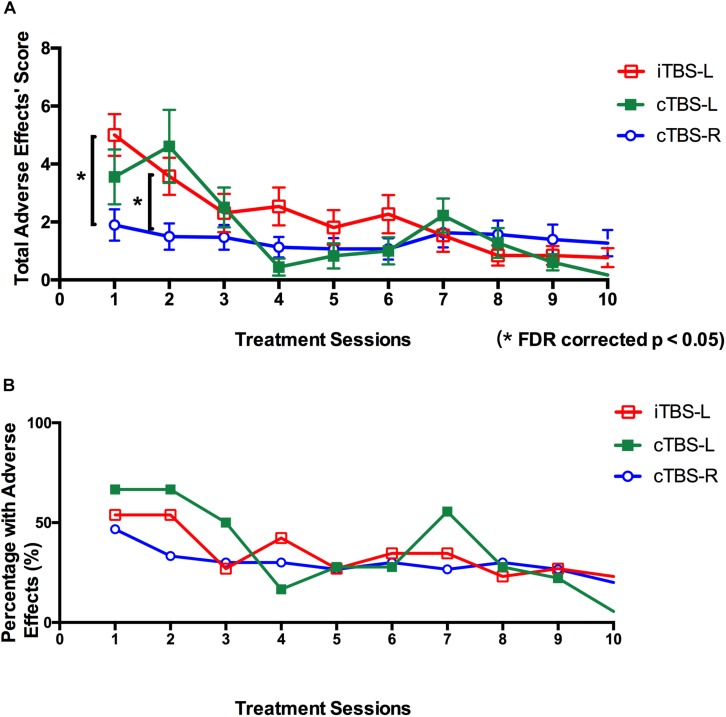
Self-reported adverse effects of twice-daily theta burst stimulation over 10 sessions. **(A)** Mean total scores of adverse effects (headache, neck pain, scalp pain, tingling, itching, burning sensations, sleepiness, trouble concentrating, and acute mood change) after each theta burst stimulation (TBS) treatment session (intermittent TBS of the left dorsolateral prefrontal cortex (iTBS-L DLPFC) (red), continuous TBS of the left DLPFC (cTBS-L DLPFC) (green), or continuous TBS of the right DLPFC (cTBS-R DLPFC) (blue). Multiple comparisons were corrected false discovery rate (FDR) correction (*P < 0.05; error bars denote SEM). **(B)** Percentage with adverse effects across all participants after each treatment session (red, iTBS-L DLPFC; green, cTBS-L DLPFC; blue, cTBS-R DLPFC).

All treatments were safe, and no seizures were reported. For the mean self-reported adverse reactions total score after each treatment, RMANOVA showed a main effect of treatment sessions (*F*_9, 657_ = 13.37, *P* < 0.001, η^2^ = 0.47) and treatment sessions × group interaction (*F*_18, 1,314_ = 3.89, *P* = 0.001, η^2^ = 0.27). Further analysis suggested that total score of adverse reactions in cTBS-R DLPFC was significantly lower than in iTBS-L DLPFC after the first treatment session (*P* = 0.01, FDR corrected) and second treatment session (*P* = 0.04, FDR corrected) (Figure 5A). As shown in Figure 5B and Table 3, the cTBS-L DLPFC group exhibited the lowest percentage (5.5%, 17 out of 18) of adverse effects after the last treatment, and the cTBS-R DLPFC and iTBS-L group demonstrated mild adverse reactions.

**TABLE 3:** Scores of adverse reactions.

	iTBS-L-D (*n* = 26)	cTBS-L-D(*n* = 18)	cTBS-R-D (*n* = 30)	ANOVA	*P* value	*Posthoc* (FDR corrected^a^)
Headache	1.88 (3.49)	3.67 (5.35)	0.57 (2.0)	*F* = 4.23	0.02	cTBS-L > iTBS-L
						cTBS-L > cTBS-R
Neck pain	0 (0)	0.11 (0.47)	0 (0)	*F* = 1.58	0.21	NA
Scalp pain	0.92 (3.30)	2.38 (4.50)	0.17 (0.53)	*F* = 3.16	0.05	cTBS-L > iTBS-L
						cTBS-L > cTBS-R
Tingling	5.35 (7.10)	5.06 (5.98)	3.90 (7.32)	*F* = 0.34	0.72	NA
Itching	0 (0)	0.11 (0.47)	0 (0)	*F* = 1.59	0.21	NA
Burning Sensation	0 (0)	0.27 (0.83)	0 (0)	*F* = 3.21	0.05	NA
Sleepiness	11.61 (18.23)	1.28 (2.49)	6.80 (15.14)	*F* = 2.69	0.08	NA
Trouble	0 (0)	1.44 (2.15)	0.233 (1.28)	*F* = 6.91	0.002	
Concentrating						cTBS-L > iTBS-L
						cTBS-L > cTBS-R
Mood Change	0.31 (1.57)	2.44 (4.68)	0.17 (0.91)	*F* = 5.19	0.008	cTBS-L > iTBS-L
						cTBS-L > cTBS-R

*Data are presented as mean (SD). ^a^Multiple comparisons were corrected by the false discovery rate (FDR) correction.*

### Blinding Effectiveness

For the self-report ratings after all treatments, Kruskal–Wallis test displayed no significant main effect of group (*P* = 0.906).

## Discussion

Adherence to treatment is a major issue in disorders of addiction. Our findings indicate the possible efficacy and tolerability of accelerated twice-daily iTBS-left or cTBS-right DLPFC treatment over 5 days in reducing craving for methamphetamine but not cTBS-left DLPFC. To our knowledge, this is the first single-blind randomized trial to systematically compare the effects of accelerated TBS procedures for methamphetamine craving targeting the DLPFC. All three interventions similarly improved mood scores, and iTBS-left TBS also improved sleep and anxiety scores, as there was no relationship with craving improvements, our craving findings may be a primary effect. Our findings converge with previous observations of efficacy of high-frequency rTMS of the left DLPFC and low-frequency rTMS of the right DLPFC to modulate craving in disorders of addictions (Li et al., 2013a; Shen et al., 2016; Terraneo et al., 2016; Yavari et al., 2016; Diana et al., 2017). Studies reporting alternate outcomes may be related to difference in TMS protocols or in efficacy as a function of the substance (Mishra et al., 2010; Höppner et al., 2011). These findings suggest that the shorter TBS procedure might serve comparably to other standard rTMS procedures in substance use disorder patients and possibly be relevant dimensionally across a range of clinical symptoms. We emphasize the need for a randomized controlled trial study with a sham control to confirm these findings.

The distribution of the percent change in the primary outcome of cue-elicited craving offered a more fine-grained comparison. We observed the highest percentage of responding, defined as a clinically relevant 50% change in craving, in the cTBS-right DLPFC group. The distributions of all three interventions were not normal, suggesting high interindividual variability to differing TBS protocols. For instance, iTBS-left DLPFC treatment may have two subgroups, with one markedly improving and a second with limited change. As this is a small sample size, further larger studies are required to address these interindividual differences in responses to neuromodulation (Li et al., 2015; Suppa et al., 2016).

TBS of the prefrontal cortex might act by enhancing aberrant prefrontal and downstream network function, decreasing aberrant excitability or plasticity or influencing downstream dopaminergic function. Methamphetamine increases synaptic dopamine levels by blocking dopamine reuptake and increasing reverse transport via the dopamine transporter. The chronic use of methamphetamine is associated with impairments in cognition, mood, and sleep (Cruickshank and Dyer, 2009); thus, normalizing mood and sleep symptoms may secondarily improve the secondary consequences of long-term amphetamine use. Chronic psychostimulants are associated with prefrontal hypofunction with impairments related to DLPFC function, including executive deficits such as working memory, planning, and goal-directed control (Goldstein and Volkow, 2011; Voon et al., 2015). TBS might thus improve DLPFC function and its associated fronto-striatal network. TMS of the DLPFC paired with functional imaging has shown a decrease in orbitofrontal activity associated nicotine cue-induced craving, particularly when the cue was immediately available, thus implicating a role in intertemporal discounting (Hayashi et al., 2013). Psychostimulants are linked to long-term downregulation of dopaminergic neurotransmission with lower D2/3 receptor levels and blunted dopamine release to psychostimulants (Volkow et al., 2004; Koob and Volkow, 2010). Methamphetamine, in particular, is associated with lower dopamine transporter levels, which can improve with abstinence (Volkow et al., 2001). The downstream influence of TMS to the DLPFC affects caudate synaptic dopamine release in healthy controls (Strafella et al., 2001) and thus may play a role in normalizing methamphetamine-related aberrant dopaminergic function. Further studies are required to assess the underlying mechanisms.

The original TBS neurophysiological study employed 80% active MT targeting the primary motor cortex (Huang et al., 2005), while other clinical trials have also tried 120% resting MT of the DLPFC demonstrating both safety and tolerability (Blumberger et al., 2018). Our previous study has shown that TBS (single session/80% motor threshold) over the motor cortex cannot induce cerebral plasticity, which indicates that neuroplasticity is supposed to be altered in methamphetamine users (Huang et al., 2017). The TBS-related plasticity and behavioral change might highly depend on the intensity, the total number of pulses, the number of sessions, and the stimulating site. In the present study, we adopted 70% resting MT intensity at DLPFC and show in this pilot study that this threshold is both effective and tolerable for most subjects. The percentage of self-reported adverse events across the sessions reduced from >50 to ∼20% within the different groups, suggesting enhanced tolerance and adaptation with repeated TBS. We show that a lower intensity (70% resting MT) for TBS might still be effective and perhaps enhance tolerability.

Treatment outcomes appeared to be differentially modulated among the three groups. Notably, the decreases in depression score were moderate (40–50%) across all protocols, suggesting a potential clinically significant response. We did not observe changes in impulsivity as measured using questionnaires. Previous studies have reported that cTBS but not iTBS of the right DLPFC reduced impulsive choice as measured using the delay-discounting task in healthy subjects and pathological gambling subjects (Cho et al., 2010; Zack et al., 2016).

Several limitations should be considered. This study focused on the effects of craving to drug cues without long-term follow-up to assess the duration of effect and impact on clinically valid outcomes such as relapse rate or the relationship to natural rewards. Whether fewer or more sessions (e.g., 8 sessions over 4 days or 20 sessions as compared to 10 sessions), a shorter interval between sessions, or a greater number of sessions per day may have a different effect remains to be investigated. The use of placebo or sham TMS in larger sample sizes would be of utility for comparison purposes, although issues have also been highlighted with the use of other forms of control groups (Davis et al., 2013). We note the larger number of dropouts in the cTBS-L DLPFC condition, thus limiting its utility as an active control; indeed, if fewer subjects had dropped out, we may have demonstrated the critical group main effect and interaction effect. Moreover, the use of neurophysiological or neuroimaging modalities would also be indicated to explore underlying mechanisms and differences underlying interindividual differences or for outcome prediction (Hawco et al., 2018). We applied iTBS over the left DLPFC but neither the right DLPFC nor a wait-list group due to technical reasons, including the limited number of methamphetamine-dependent subjects who could be recruited and the length of the experiment. The clinical effects of iTBS over the right DLPFC and a wait-list group in methamphetamine-dependent subjects should be further investigated. Finally, given that patients were recruited from an ongoing rehabilitation center training program, the findings should be interpreted with caution since daily physical exercise and supportive therapy or individual psychological therapy might alter sleep or mood status. However, crucially, subjects across all groups experienced the same non-TBS-related interventions, and we further show that the improvements in mood and sleep were unrelated to the improvement in craving.

## Conclusion

Our findings add to the growing evidence that accelerated TBS might be an efficacious method for craving, mood, sleep, and anxiety symptoms and tolerability in abstinent methamphetamine-dependent subjects. Further larger randomized studies with placebo control and comparisons with standard TBS or standard rTMS protocols are indicated. Critically, our results suggest that the use of both TBS and an accelerated design might show efficacy in targeting methamphetamine craving and emphasize efficiency, potentially facilitating the number of patients that can be treated with each TMS machine and shortening the duration of treatment from several weeks to 1 week.

## Data Availability Statement

The datasets generated for this study are available on request to the corresponding author.

## Ethics Statement

The studies involving human participants were reviewed and approved by the research ethics boards of Shanghai Mental Health Center, Nanjing Normal University and the local safety monitoring board (Chinese Clinical Trial Registry number, ChiCTR-INR-17013610). The patients/participants provided their written informed consent to participate in this study.

## Author Contributions

T-FY and VV conceptualized and designed the study. DZ, YL, and TL performed the study. DZ, VV, and T-FY analyzed the results and wrote the manuscript together. All authors have read and approved the final version of the manuscript.

## Conflict of Interest

The authors declare that the research was conducted in the absence of any commercial or financial relationships that could be construed as a potential conflict of interest.

## References

[B1] Amiaz R. Levy D. Vainiger D. Grunhaus L. Zangen A. (2009). Repeated high−frequency transcranial magnetic stimulation over the dorsolateral prefrontal cortex reduces cigarette craving and consumption. *Addiction* 104 653–660. 10.1111/j.1360-0443.2008.02448.x 19183128

[B2] Bakker N. Shahab S. Giacobbe P. Blumberger D. M. Daskalakis Z. J. Kennedy S. H. (2015). Rtms of the dorsomedial prefrontal cortex for major depression: safety, tolerability, effectiveness, and outcome predictors for 10 hz versus intermittent theta-burst stimulation. *Brain Stimul.* 8 208–215. 10.1016/j.brs.2014.11.002 25465290

[B3] Beck A. T. Epstein N. Brown G. Steer R. A. (1988). An inventory for measuring clinical anxiety: psychometric properties. *J. Consult. Clin. Psychol.* 56 893–897. 10.1037/0022-006x.56.6.893 3204199

[B4] Beck A. T. Ward C. H. Mendelson M. Mock J. Erbaugh J. (1961). An inventory for measuring depression. *Arch. Gen. Psychiatry* 4 561–571. 13688369 10.1001/archpsyc.1961.01710120031004

[B5] Benjamini Y. Yekutieli D. (2001). The control of the false discovery rate in multiple testing under dependency. *Annals Stat.* 29 1165–1188. 10.1186/1471-2105-9-114 18298808 PMC2375137

[B6] Blumberger D. M. Vila-Rodriguez F. Thorpe K. E. Feffer K. Noda Y. Giacoble P. (2018). Effectiveness of theta burst versus high-frequency repetitive transcranial magnetic stimulation in patients with depression (three-d): a randomised non-inferiority trial. *Lancet* 391 1683–1692. 10.1016/S0140-6736(18)30295-2 29726344

[B7] Buysse D. J. Reynolds C. F. III Monk T. H. Berman S. R. Kupfer D. J. (1989). The pittsburgh sleep quality index: a new instrument for psychiatric practice and research. *Psychiatry Res.* 28 193–213. 10.1016/0165-1781(89)90047-4 2748771

[B8] Camprodon J. A. Martínez-Raga J. Alonso-Alonso M. Shih M.-C. Pascual-Leone A. (2007). One session of high frequency repetitive transcranial magnetic stimulation (rtms) to the right prefrontal cortex transiently reduces cocaine craving. *Drug Alcohol Depend.* 86 91–94. 10.1016/j.drugalcdep.2006.06.002 16971058

[B9] Cho S. S. Ko J. H. Pellecchia G. Van Eimeren T. Cilia R. Strafella A. P. (2010). Continuous theta burst stimulation of right dorsolateral prefrontal cortex induces changes in impulsivity level. *Brain Stimul.* 3 170–176. 10.1016/j.brs.2009.10.002 20633446 PMC3707839

[B10] Cruickshank C. C. Dyer K. R. (2009). A review of the clinical pharmacology of methamphetamine. *Addiction* 104 1085–1099. 10.1111/j.1360-0443.2009.02564.x 19426289

[B11] Davis N. J. Gold E. Pascual-Leone A. Bracewell R. M. (2013). Challenges of proper placebo control for non-invasive brain stimulation in clinical and experimental applications. *Eur. J. Neurosci.* 38 2973–2977. 10.1111/ejn.12307 23869660

[B12] Di Lazzaro V. Pilato F. Saturno E. Oliviero A. Dileone M. Mazzone P. (2005). Theta-burst repetitive transcranial magnetic stimulation suppresses specific excitatory circuits in the human motor cortex. *J. Physiol.* 565 945–950. 10.1113/jphysiol.2005.087288 15845575 PMC1464561

[B13] Diana M. Raij T. Melis M. Nummenmaa A. Leggio L. Bonci A. (2017). Rehabilitating the addicted brain with transcranial magnetic stimulation. *Nat. Rev. Neurosci.* 18 685–693. 10.1038/nrn.2017.113 28951609

[B14] Dinur-Klein L. Dannon P. Hadar A. Rosenberg O. Roth Y. Kotler M. (2014). Smoking cessation induced by deep repetitive transcranial magnetic stimulation of the prefrontal and insular cortices: A prospective, randomized controlled trial. *Biol. Psychiatry* 76 742–749. 10.1016/j.biopsych.2014.05.020 25038985

[B15] Everitt B. J. Robbins T. W. (2016). Drug addiction: Updating actions to habits to compulsions ten years on. *Annu. Rev. Psychol.* 67 23–50. 10.1146/annurev-psych-122414-033457 26253543

[B16] George M. S. Stallings L. E. Speer A. M. Nahas Z. Spicer K. M. Vincent D. J. (1999). Prefrontal repetitive transcranial magnetic stimulation (rtms) changes relative perfusion locally and remotely. *Human Psychopharmacol. Clin. Exp.* 14 161–170. 10.1002/(sici)1099-1077(199904)14:3<161::aid-hup73>3.0.co;2-2

[B17] Goldstein R. Z. Volkow N. D. (2011). Dysfunction of the prefrontal cortex in addiction: neuroimaging findings and clinical implications. *Nat. Rev. Neurosci.* 12 652–669. 10.1038/nrn3119 22011681 PMC3462342

[B18] Gordon H. W. (2016). Laterality of brain activation for risk factors of addiction. *Curr. Drug Abuse Rev.* 9 1–18. 10.2174/1874473709666151217121309 26674074 PMC4811731

[B19] Hanlon C. A. Kearney-Ramos T. Dowdle L. T. Hamilton S. DeVries W. Mithoefer O. (2017). Developing repetitive transcranial magnetic stimulation (rtms) as a treatment tool for cocaine use disorder: a series of six translational studies. *Curr. Behav. Neurosci. Rep.* 4 341–352. 10.1007/s40473-017-0135-4 30009124 PMC6039979

[B20] Hawco C. Voineskos A. N. Steeves J. K. E. Dickie E. W. Viviano J. D. Downar J. (2018). Spread of activity following tms is related to intrinsic resting connectivity to the salience network: A concurrent tms-fmri study. *Cortex* 108 160–172. 10.1016/j.cortex.2018.07.010 30195825

[B21] Hayashi T. Ko J. H. Strafella A. P. Dagher A. (2013). Dorsolateral prefrontal and orbitofrontal cortex interactions during self-control of cigarette craving. *Proc. Natl. Acad. Sci. U.S.A.* 110 4422–4427. 10.1073/pnas.1212185110 23359677 PMC3600476

[B22] Herwig U. Satrapi P. Schönfeldt-Lecuona C. (2003). Using the international 10-20 eeg system for positioning of transcranial magnetic stimulation. *Brain Topogr.* 16 95–99. 10.1023/B:BRAT.0000006333.93597.9d 14977202

[B23] Höppner J. Broese T. Wendler L. Berger C. Thome J. (2011). Repetitive transcranial magnetic stimulation (rtms) for treatment of alcohol dependence. *World J. Biol. Psychiatry* 12 57–62. 10.3109/15622975.2011.598383 21905997

[B24] Huang X. Chen Y.-Y. Shen Y. Cao X. Li A. Liu Q. (2017). Methamphetamine abuse impairs motor cortical plasticity and function. *Mol. Psychiatry* 22, 1274–1281. 10.1038/mp.2017.143 28831198 PMC5582165

[B25] Huang Y.-Z. Edwards M. J. Rounis E. Bhatia K. P. Rothwell J. C. (2005). Theta burst stimulation of the human motor cortex. *Neuron* 45 201–206. 15664172 10.1016/j.neuron.2004.12.033

[B26] Kammer T. Beck S. Thielscher A. Laubis-Herrmann U. Topka H. (2001). Motor thresholds in humans: a transcranial magnetic stimulation study comparing different pulse waveforms, current directions and stimulator types. *Clin. Neurophysiol.* 112 250–258. 10.1016/S1388-2457(00)00513-7 11165526

[B27] Koob G. F. Volkow N. D. (2010). Neurocircuitry of addiction. *Neuropsychopharmacology* 35:217. 10.1038/npp.2009.110 PMC2805560 19710631

[B28] Li L. M. Uehara K. Hanakawa T. (2015). The contribution of interindividual factors to variability of response in transcranial direct current stimulation studies. *Front. Cell. Neurosci.* 9:181. 10.3389/fncel.2015.00181 26029052 PMC4428123

[B29] Li X. Hartwell K. J. Owens M. Lematty T. Borckardt J. J. Hanlon C. A. (2013a). Repetitive transcranial magnetic stimulation of the dorsolateral prefrontal cortex reduces nicotine cue craving. *Biol. Psychiatry* 73 714–720. 10.1016/j.biopsych.2013.01.003 23485014 PMC3615051

[B30] Li X. Malcolm R. J. Huebner K. Hanlon C. A. Taylor J. J. Brady K. T. (2013b). Low frequency repetitive transcranial magnetic stimulation of the left dorsolateral prefrontal cortex transiently increases cue-induced craving for methamphetamine: a preliminary study. *Drug Alcohol Depend.* 133 641–646. 10.1016/j.drugalcdep.2013.08.012 24028801 PMC4196687

[B31] Lopez-Alonso V. Cheeran B. Rio-Rodriguez D. Fernandez-Del-Olmo M. (2014). Inter-individual variability in response to non-invasive brain stimulation paradigms. *Brain Stimul.* 7 372–380. 10.1016/j.brs.2014.02.004 24630849

[B32] McGirr A. Van den Eynde F. Tovar-Perdomo S. Fleck M. P. A. Berlim M. T. (2015). Effectiveness and acceptability of accelerated repetitive transcranial magnetic stimulation (rtms) for treatment-resistant major depressive disorder: An open label trial. *J. Affect. Disord.* 173 216–220. 10.1016/j.jad.2014.10.068 25462419

[B33] Mishra B. R. Nizamie S. H. Das B. Praharaj S. K. (2010). Efficacy of repetitive transcranial magnetic stimulation in alcohol dependence: a sham−controlled study. *Addiction* 105 49–55. 10.1111/j.1360-0443.2009.02777.x 20078462

[B34] Modirrousta M. Meek B. P. Wikstrom S. L. (2018). Efficacy of twice-daily vs once-daily sessions of repetitive transcranial magnetic stimulation in the treatment of major depressive disorder: a retrospective study. *Neuropsychiatr. Dis. Treat* 14 309–316. 10.2147/NDT.S151841 29398915 PMC5775741

[B35] Politi E. Fauci E. Santoro A. Smeraldi E. (2008). Daily sessions of transcranial magnetic stimulation to the left prefrontal cortex gradually reduce cocaine craving. *Am. J. Addict.* 17 345–346. 10.1080/10550490802139283 18612892

[B36] Reise S. P. Moore T. M. Sabb F. W. Brown A. K. London E. D. (2013). The barratt impulsiveness scale-11: reassessment of its structure in a community sample. *Psychol. Assess.* 25 631–642. 10.1037/a0032161 23544402 PMC3805371

[B37] Rose J. E. McClernon F. J. Froeliger B. Behm F. M. Preud’homme X. Krystal A. D. (2011). Repetitive transcranial magnetic stimulation of the superior frontal gyrus modulates craving for cigarettes. *Biol. Psychiatry* 70 794–799. 10.1016/j.biopsych.2011.05.031 21762878

[B38] Sanna A. Fattore L. Badas P. Corona G. Cocco V. Diana M. (2019). Intermittent theta burst stimulation of the prefrontal cortex in cocaine use disorder: a pilot study. *Front. Neurosci.* 13:765. 10.3389/fnins.2019.00765 31402851 PMC6670008

[B39] Schulze L. Feffer K. Lozano C. Giacobbe P. Daskalakis Z. J. Blumberger D. M. (2018). Number of pulses or number of sessions? an open-label study of trajectories of improvement for once-vs. Twice-daily dorsomedial prefrontal rtms in major depression. *Brain Stimul.* 11 327–336. 10.1016/j.brs.2017.11.002 29153439

[B40] Sheffer C. E. Bickel W. K. Brandon T. H. Franck C. T. Deen D. Panissidi L. (2018). Preventing relapse to smoking with transcranial magnetic stimulation: feasibility and potential efficacy. *Drug Alcohol Depend.* 182 8–18. 10.1016/j.drugalcdep.2017.09.037 29120861 PMC5836507

[B41] Shen Y. Cao X. Tan T. Shan C. Wang Y. Pan J. (2016). 10-hz repetitive transcranial magnetic stimulation of the left dorsolateral prefrontal cortex reduces heroin cue craving in long-term addicts. *Biol. Psychiatry* 80 e13–e14. 10.1016/j.biopsych.2016.02.006 26995024

[B42] Steele V. Maxwell A. M. Ross T. Stein E. Salmeron B. J. (2019). Accelerated intermittent theta-burst stimulation as a treatment for cocaine use disorder: a proof-of-concept study. *Front. Neurosci.* 13:1147. 10.3389/fnins.2019.01147 31736689 PMC6831547

[B43] Strafella A. P. Paus T. Barrett J. Dagher A. (2001). Repetitive transcranial magnetic stimulation of the human prefrontal cortex induces dopamine release in the caudate nucleus. *J. Neurosci.* 21 RC157–RC157. 11459878 10.1523/JNEUROSCI.21-15-j0003.2001 PMC6762641

[B44] Su H. Zhong N. Gan H. Wang J. Han H. Chen T. (2017). High frequency repetitive transcranial magnetic stimulation of the left dorsolateral prefrontal cortex for methamphetamine use disorders: a randomised clinical trial. *Drug Alcohol Depend.* 175 84–91. 10.1016/j.drugalcdep.2017.01.037 28410525

[B45] Suppa A. Huang Y. Z. Funke K. Ridding M. C. Cheeran B. Di Lazzaro V. (2016). Ten years of theta burst stimulation in humans: established knowledge, unknowns and prospects. *Brain Stimul.* 9 323–335. 10.1016/j.brs.2016.01.006 26947241

[B46] Terraneo A. Leggio L. Saladini M. Ermani M. Bonci A. Gallimberti L. (2016). Transcranial magnetic stimulation of dorsolateral prefrontal cortex reduces cocaine use: a pilot study. *Eur. Neuropsychopharmacol.* 26 37–44. 10.1016/j.euroneuro.2015.11.011 26655188 PMC9379076

[B47] Volkow N. D. Chang L. Wang G.-J. Fowler J. S. Franceschi D. Sedler M. (2001). Loss of dopamine transporters in methamphetamine abusers recovers with protracted abstinence. *J. Neurosci.* 21 9414–9418. 10.1523/jneurosci.21-23-09414.2001 11717374 PMC6763886

[B48] Volkow N. D. Fowler J. S. Wang G.-J. Swanson J. M. (2004). Dopamine in drug abuse and addiction: results from imaging studies and treatment implications. *Mol. Psychiatry* 9:557. 10.1038/sj.mp.4001507 15098002

[B49] Voon V. Derbyshire K. Rück C. Irvine M. A. Worbe Y. Enander J. (2015). Disorders of compulsivity: a common bias towards learning habits. *Mol. Psychiatry* 20:345. 10.1038/mp.2014.44 24840709 PMC4351889

[B50] Yavari F. Shahbabaie A. Leite J. Carvalho S. Ekhtiari H. Fregni F. (2016). Noninvasive brain stimulation for addiction medicine: from monitoring to modulation. *Prog. Brain Res.* 224 371–399. 10.1016/bs.pbr.2015.08.007 26822367

[B51] Zack M. Cho S. S. Parlee J. Jacobs M. Li C. Boileau I. (2016). Effects of high frequency repeated transcranial magnetic stimulation and continuous theta burst stimulation on gambling reinforcement, delay discounting, and stroop interference in men with pathological gambling. *Brain Stimul.* 9 867–875. 10.1016/j.brs.2016.06.003 27350401

[B52] Zhang J. J. Fong K. N. Ouyang R. G. Siu A. M. Kranz G. S. (2019). Effects of repetitive transcranial magnetic stimulation (rtms) on craving and substance consumption in patients with substance dependence: a systematic review and meta−analysis. *Addiction* 114 2137–2149. 10.1111/add.14753 31328353

